# Psychological Counseling Service “Together” at University of Genoa: Students’ Psychological Profile in Pre and Post Pandemic

**DOI:** 10.3389/fpsyg.2022.898530

**Published:** 2022-05-27

**Authors:** Cecilia Serena Pace, Maria Carmen Usai, Fabiola Bizzi, Patrizia Minetto, Alberta Alcetti, Mirella Zanobini

**Affiliations:** Department of Educational Sciences, University of Genoa, Genoa, Italy

**Keywords:** psychological counseling, university students, COVID-19 pandemic, emotional dysregulation, pathological personality domains, learning believes, anxiety

## Abstract

The present explorative study aims to analyze the profiles of students seeking help in the two areas (emotion/relation or learning areas) of a psychological counseling service for students at the University of Genoa to better understand their request for support in pre- and post-pandemic periods. A total of 229 university students seeking for help from November 2018 to December 2021 completed a psychological battery investigating emotion regulation difficulties and pathological domains of personality (students taken in charge by the emotion/relation area) or motivation issues and anxiety and resilience levels (students taken in charge by the learning area). Regarding the emotion/relation area, results show that problems in emotion dysregulation, and especially in regulating positive emotions, are associated to several pathological domains of personality, such as Psychoticism, Antagonism, Disinhibition, Detachment, and Negative Affectivity. Among the learning area, motivational aspects concerning confidence in one’s intelligence, academic self-efficacy, and learning goals are differently associated with anxiety and resilience above and beyond other aspects, such as attributions. Some limited but significant differences emerge from the comparison between pre- and post-COVID periods: a reduction in detachment in students attending the emotion/relation area, an increase of students with high levels of anxiety in the learning area. These results support the importance of continually operating on emotional and motivational aspects to enhance the students’ well-being and thus sustaining their academic careers.

## Introduction

Psychological counseling is defined “*as the professional use, regulated by principles, of a relationship, in which the client is helped to acquire a better knowledge of himself/herself and of his/her own psychological problems, and to sustain his/her emotional growth and the optimal development of his/her own personal resources*” (Adamo et al., 2010, p. 1152). Specifically, university counseling services deal with students’ psychological problems concerning the challenge of emerging adulthood, and the developmental transitioning they are facing with the addition of issues related to adaptation to the new academic life. Recently, the COVID-19 pandemic posed a double challenge for university psychological services: on one hand, there has been a noticeable increase in demands for psychological help from university students’ (Karaman et al., 2021; Muzi et al., 2021); on the other hand, university counseling services were obliged to move from presence (usual) to distance mode of delivery to ensure continuity of services (Supriyanto et al., 2020), as consequence of restrictions on face-to-face social contacts imposed in several countries, starting in Italy from March 2020.

Most studies on psychological profiles of pre-pandemic seeking-help university students focused on their mental health issues – i.e., rates of depression, anxiety, suicidality, substance abuse, etc., – as well as on their associated poor academic achievements, and some studies investigated which risk factors can be related with these negative outcomes (for a review, see Sheldon et al., 2021). Among these, emotion dysregulation and pathological domains of personality, as well as causal attribution and motivational aspects deserve to be further explored.

### Emotion Dysregulation and Pathological Personality Among University Students

Emotion dysregulation is a multi-faceted construct involving deficits in understanding, responding to, and management of emotional responses (Gratz and Roemer, 2004). Literature has focused on dysregulation stemming from negative emotions, highlighting that the impairments in regulating unpleasant emotions -as fear, sadness, anger, related to self-reported stress, and poor coping- are extremely linked to maladaptive behaviors and psychopathologies, including personality disorders (Ponzoni et al., 2021). Focusing on university students, research showed that those experiencing negative affect and difficulties in regulating those intense emotions often display interpersonal conflict, aggression, and symptomatology (Menozzi et al., 2016).

However, preliminary work suggests that individuals experience difficulties regulating “positive” emotions (i.e., pleasure emotions as excitement, joy, and happiness) are parallel to the difficulties observed in negative emotions (Weiss et al., 2018). Indeed, positive emotional states may increase distractibility and lead to less discriminative use of information increasing the risk for disadvantageous decision-making focused on short- versus long-term goals and interfering with the ability to control impulses (Weiss et al., 2018). However, no study investigated dysregulation of positive emotions in high-educated students up to now.

Furthermore, clinical research has recently focused on pathological personality not only in terms of categorical personality disorders, but also in terms of dimensional traits, i.e., maladaptive variants of the five domains of the extensively validated Big Five personality model (Thomas et al., 2013). It includes: Psychoticism (i.e., a disconnection from reality and a tendency for illogical thought patterns), Antagonism (i.e., aggressive tendencies accompanied by assertions of dominance and grandiosity), Disinhibition (i.e., characterized by impulsivity and sensation seeking), Detachment (i.e., characterized by introversion, social isolation, and anhedonia), and Negative Affectivity (i.e., the tendency to experience an array of negative emotions). Literature showed that pathological personality traits can contribute to the development of health problems (Abdi and Pak, 2019). For example, studies conducted with high-educated students highlighted that pathological personality traits are associated with anxiety-related conditions, as well as social interactions problems (Zemestani et al., 2021). These results are consistent with the idea that certain pathological personality traits are associated with difficulties to regulate the emotions and negative outcomes as interpersonal problems, and aggression (Aldao et al., 2010; Pollock et al., 2016). This is important because the present study may shed light on the role that emotion regulation difficulties and pathological personality traits may play in university context.

Considering the COVID pandemic, both emotion dysregulation and pathological personality traits displayed a less favorable pattern of outcomes with a general worsening of individual mental health (Zemestani et al., 2021; Waterschoot et al., 2022). However, few studies have examined these aspects among university students, although they may be crucial factors for maladjustment in the university context having implications for the well-being of students, their future personal development, and also for their academic performance (Dobos et al., 2021).

### Causal Attribution, Motivational Aspects, and Learning Difficulties Among University Students

The literature shows that motivational and strategic aspects of learning positively modulate a successful career at university (Cornoldi et al., 2003; Dupeyrat and Mariné, 2005). Accordingly, different motivational aspects, such as learning goals, self-efficacy, and confidence in our-own intelligence may be considered relevant for optimal university performance. For example, learning goals orientation leads students to approach, engage and respond to learning tasks in specific ways (Schunk et al., 2008). Being guided by mastery goals has positive effects on learning (Denzine and Brown, 2015): students who show mastery goals tend to worry less about comparing their own with the performance of others, set goals related to themselves, and put more effort into pursuing them. The goals orientation is also associated with other motivational aspects, thus mastery-oriented students tend to show higher levels of self-efficacy, which is the individual’s perception of their ability to complete a task, that in turn promotes performance (Bandura, 1997). Together with these aspects, the causal attributions on academic results influence students’ attitudes toward subsequent learning opportunities, constituting key beliefs within motivational processes (Weiner, 1985). Thus, the tendency to attribute one’s successes and failures to internal causes, such as commitment or ability, or to external causes, such as luck or the characteristics of a task, may change the individual reactions in face of academic failures or successes.

Considering the role played by the aforementioned aspects on the learning process, students who have applied to the counseling service reporting difficulties in studying receive an intervention aimed at promoting the motivational aspects and beliefs associated with an improvement in academic results, also with the aim of reducing the study’s related negative emotions that entail an increase of anxiety.

#### Current Study

Based on the abovementioned literature, the current explorative study has the following objectives.

The first purpose was to examine the pattern of correlations among emotion regulation difficulties and pathological personality domains. It was hypothesized that dysregulation of both negative emotions and positive emotions (still unexplored) would be positively related to pathological personality domains as Psychoticism, Antagonism, Disinhibition, Detachment, and Negative Affectivity (Pollock et al., 2016). It was also verified whether seeking-help university students in the post-pandemic condition would show increased levels of emotion dysregulation and pathological personality domains compared to those in pre-pandemic conditions (Zemestani et al., 2021; Waterschoot et al., 2022).

The second purpose is to examine the pattern of correlations among motivational variables and between them and the outcome variables: anxiety and resilience. It was hypothesized that anxiety would be negatively related, and resilience would be positively related to students’ self-efficacy, confidence on their own intelligence, and mastery-oriented learning goals. Furthermore, it was verified whether students in the post COVID condition would show an increased level of anxiety and a reduced resilience compared to those in pre-pandemic conditions.

## Methods

### Procedure

*Psychological counseling service at University of Genoa: “Together.”* The current study involved students who sought help to “Together,” a counseling service or higher-educated students that has been established at the University of Genoa since the end of 2018. All students enrolled at the University of Genoa can freely accede to the service by completing a form that allows them to be assigned to one of two intervention areas, one for emotional-relational difficulties (emotion/relation area, from now ERA) and one for learning difficulties (learning area, from now LA). The counseling process comprises a cycle of five sessions plus a follow-up after 3 months. The counseling service is carried out by two psychologists–psychotherapists, one psychoanalytically oriented (ERA) and one with a cognitive-behavioral approach (LA), postgraduate trainees in psychology, and academic researchers. Until COVID-19 pandemic (March 2020), the counseling interviews at “Together” were delivered in presence in a dedicated room at University of Genoa. As a result of the restrictions due to the pandemic, from April 2020 “Together” switched to digital mode and distance counseling services were offered, to continue helping students.

With regard to the study’s procedure, during the first meeting, students were asked to voluntarily fill in the assessment tools described below in the section “Materials,” specific for each area, ERA and LA, and those who agreed to participate in the research signed the informed consent in line with the recommendations of the Ethical Code of the National Council of Psychologists and the Ethical Guidelines of the Italian Psychology Association. Data from these tools is used in two different ways: clinicians in each area use individual data to work clinically with the client in the counseling process, while researchers anonymized and statistically analyzed aggregated data to get a picture of psychological profiles of college students seeking for counseling.

### Participants

University students who sought help to the “Together” were enrolled in this study. Data of 229 participants taken in charge from November 2018 are presented (see Table 1). Out of them, 128 were taken into charge by the ERA and 101 by the LA.

**TABLE 1:** Sample description (*N* = 229).

Service areas	N. total	Gender % females	Mean age (S.D.)	Age range, years	N. Post-COVID accesses
Emotion/relation area (ERA)	128	73	22.84 (2.84)	18–33	87
Learning area (LA)	101	64	23.5 (2.75)	19–33	61
All “Together”	229	70	23.17 (3.8)	18–33	148

*N., number; S.D., standard deviation.*

Among participants, 148 students requested the Service from March 2020 to December 2021, and they were considered as post-pandemic accesses. As regards the differences between pre- and post-pandemic accesses, it is worth noting an estimated increase of about 34% between the periods before and after March 2020, with 5 and 6.7 monthly admissions, respectively.

### Materials

#### Emotion/Relation Area Measures

*Difficulties in Emotion Regulation Scale* (DERS; Giromini et al., 2012) is a 36-item self-report measure assessing emotion regulation problems using a Likert-type scale rated from 1 (almost never) to 5 (almost always). It provides a Total Score (DERS Total) resulting from the sum of six additional subscales: (1) non-acceptance of negative emotions (Non-acceptance); (2) inability to conduct targeted behaviors when experiencing negative emotions (Goals); (3) difficulty controlling impulsive behaviors when experiencing negative emotions (Impulse); (4) limited access to regulating strategies that are deemed effective (Strategies); (5) lack of awareness of one’s emotions (Awareness); and (6) lack of understanding of the nature of one’s emotional responses (Clarity). DERS Total Cronbach’s α = 0.92.

*Difficulties in Emotion Regulation Scale-Positive* (DERS-P; Velotti et al., 2020) is a 15-item self-report measure developed to assess clinically relevant difficulties in the regulation of positive emotions, that uses a 5-point Likert scale ranging from 1 (almost never) to 5 (almost always). It provides a Total Score (DERS-P Total) resulting from the sum of three additional subscales: (a) acceptance of positive emotions (Non-acceptance); (b) ability to engage in goal-directed behavior when experiencing positive emotions (Goals); and (c) ability to control impulsive behaviors when experiencing positive emotions (Impulse). Higher scores indicate greater difficulties in the regulation of positive emotions. Rather than beginning with the phrase “When I’m upset” like many of the original DERS items, the DERS-Positive items begin with the phrase “When I’m happy.” DERS-P Total Cronbach’s α = 0.86.

*Personality Inventory for DSM-5* (PID-5; Fossati et al., 2013) is a 220-item questionnaire with a 4-point response scale (0 = very or often false to 3 = very or often true) designed to assess the personality traits of the alternative model for personality disorders based on personality dysfunction and pathological personality traits introduced with publication of the DSM-5. PID-5 items (e.g., “I worry a lot about being alone”) are summed to compose PID-5 trait scale scores, and 5 domain scale scores (Psychoticism, Antagonism, Disinhibition, Detachment, and Negative Affectivity). Cronbach’s α ranges from 0.72 to 0.87.

#### Learning Area Measures

*Attributional Style Questionnaire* (ASQ; De Beni and Moé, 1995) was used to measure the individual attributions of success and failure in cognitive tasks. It comprises 24 items presenting successful (12) or unsuccessful (12) hypothetical situations, in which the student may have found himself. Participants had to select two causal explanations out of five indicating effort, ability, luck, help, or task characteristics. Cronbach’s α = 0.67.

The students were also asked to rate two self-reports from the AMOS questionnaires (De Beni et al., 2014): three subscales from the *Beliefs Questionnaire* (BQ), that measure motivational aspects, such as confidence in our-own intelligence, academic self-efficacy, and being focused on performance or mastery learning goals. Students were asked to answer 12 items on a six/five-point Likert scale, organized into the intelligence self-confidence (ISC, 3 items), the Academic Self-efficacy (ASE, 5 items), and the Learning goals (LG, 4 items) subscales. Cronbach’s alphas for each scale were: 0.66 for ISC, 0.80 for ASE, and 0.78 for LG; the *Anxiety and Resilience Questionnaire* consists of 14 items in a five-point Likert scale measuring anxiety (7 items) and resilience (7 items). High scores correspond to higher anxiety levels and high resilience levels. The Cronbach’s alphas are 0.86 and 0.76 for anxiety and resilience subscales.

### Statistics

Descriptive statistics were reported to describe participants’ demographics. Zero-order Person correlation coefficients were used to analyze correlations among variables. ANOVAs were performed to compare pre- and post-pandemic groups in all the study variables.

## Results

Results are separately presented for each area.

### Emotion/Relation Area: Relations Between Emotion Dysregulation and Pathological Domains of Personality, and Impact of Pandemic

As shown in Table 2, difficulties in both negative and positive emotion regulation are positively correlated with Psychoticism, Disinhibition, and Negative Affectivity. Moreover, both Antagonism and Detachment are positively correlated only with difficulties in the regulation of positive emotions.

**TABLE 2:** ERA: Descriptive statistics (*N* = 128) and zero-order correlation (Pearson).

	Mean	S.D.	Min	Max	Age	DERS total	DERS-P total
DERS Total	100.60	21.99	55	158	0.94		
DERS-P Total	17.45	5.81	0	42	0.70	0.28**	
PID 5 Psychoticism	0.81	0.42	0.00	2.15	0.33	0.34***	0.42***
PID 5 Antagonism	0.61	0.41	0.00	1.79	0.14	0.13	0.24**
PID 5 Disinhibition	1.00	0.36	0.15	2.18	0.77	0.42***	0.35***
PID 5 Detachment	1.26	0.50	0.25	2.59	0.17	0.16	0.21*
PID 5 Negative Affectivity	1.16	0.40	0.05	2.03	0.26	0.34***	0.41***

*DERS, Difficulties in Emotion Regulation Scale; DERS-P, Difficulties in Emotion Regulation Scale-Positive; PID-5, Personality Inventory for DSM-5; S.D., standard deviation.*

*p < 0.05; ** <0.01; *** <0.001.

The ANOVAs exploring differences in negative and positive emotion regulation difficulties and pathological domains of personality associated with the pandemic period reveal only a significant effect concerning PID-5 Detachment [*F*(1,123) = 3.74, *p* = 0.000, η^2^*p* = 0.24].

### Learning Area: Relations Between Attributions, Anxiety and Resilience, and Impact of Pandemic

As shown in Table 3, internal attributions on effort and ability are both negatively associated with the other external causal attributions. Internal causes did not correlate between them, nor did external causes result associated with each other. As regards to the association between attributions and beliefs, effort is positively correlated with intelligence self-confidence and learning goals, while luck is negatively associated with intelligence self-confidence. As considering the pattern of correlation with the outcomes, anxiety is negatively associated with intelligence self-confidence, academic self-efficacy, and learning goals, while resilience is positively associated with academic self-efficacy. Anxiety is negatively correlated with resilience.

**TABLE 3:** LA: Descriptive statistics (*N* = 101) and zero-order correlation (Pearson).

	*N*	Mean	S.D.	Min	Max	Age	Effort	Ability	Task	Luck	Help	ISC	ASE	*LG*	Anxiety
Effort	91	–0.63	1.10	−3.71	2.15	0.06	−								
Ability	91	0.34	0.76	−1.25	3.29	0.08	–0.03	−							
Task	91	–0.31	0.90	−3.04	2.10	0.03	−0.25*	−0.30**	−						
Luck	90	0.36	0.86	−1.65	2.70	–0.11	−0.55***	−0.24*	–0.05	−					
Help	88	0.25	0.85	−1.23	2.75	0.02	−0.32**	−0.22*	0.01	0.03	−				
ISC	87	–0.37	1.11	−6.03	1.39	0.07	0.34**	0.04	–0.02	−0.29*	–0.08	−			
ASE	87	–0.87	1.32	−4.89	2.20	0.07	0.18	–0.06	0.02	–0.09	–0.04	0.34**	−		
LG	86	–0.31	1.22	−2.30	1.40	0.18	0.24*	–0.05	–0.13	–0.15	0.12	0.22*	0.21	−	
Anxiety	85	3.19	1.83	−1.35	6.44	0.04	–0.18	–0.001	0.08	0.19	–0.16	−0.43***	−0.24*	−0.30**	
Resilience	85	–1.09	1.22	−4.05	3.22	–0.09	0.15	–0.04	0.03	–0.15	–0.01	0.27*	0.49***	0.23	−0.32**

*S.D., standard deviation; ISC, Intelligence Self-Confidence; ASE, Academic Self-Efficacy; LG, Learning Goals.*

*p < 0.05; ** <0.01; ***p < 0.001.

The two ANOVAs exploring differences in anxiety and resilience associated with the pandemic period did not reveal any significant effect [anxiety *F*(1,83) = 2.07, *p* = 0.154, η^2^*p* = 0.024; resilience *F*(1,83) = 0.101, *p* = 0.752, η^2^*p* = 0.001]. However, considering the number of students who show critical scores in the anxiety and resilience scales (higher or equal to 1.5 S.D. and lower or equal to −1.5 S.D., respectively), there is a significant increase of students who show critical levels of anxiety during the pandemic period, while the number of students with lower levels of resilience did not change significantly: 21 pre- and 48 post-pandemic accesses with high anxiety (χ^2^ = 10.6, *p* = 0.001), vs. 15 pre- and 21 post-pandemic accesses with low resilience (χ^2^ = 1, *p* = 0.317).

## Discussion

The hypotheses concerning the ERA were partially confirmed. Indeed, dysregulation in negative emotions was positively correlated with specific pathological personality domains as Psychoticism, Disinhibition, and Negative Affectivity, but not with Antagonism, and Detachment. In line with the literature (Pollock et al., 2016), these findings suggest that high-educated students with difficulties to regulate negative emotions could respond to academic performance with heightened feelings of tension, anxiety, fear, impulsivity or illogical thoughts, thus interfering to their adjustment, plan the behaviors and projects into the future (Dobos et al., 2021). Otherwise, difficulties in regulating negative emotion were not associated with the avoidance of socioemotional experiences and with behaviors that put the individual at odds with other people, therefore other certain emotion difficulties could come into play to better understand the individual functioning difficulties experimented in university context. On the contrary, dysregulation in positive emotions was positively correlated with all the five pathological domains of personality, suggesting a key-role of positive emotions dysregulation as a crucial factor for maladjustment in the university context. This sheds light that dysregulation of positive emotions can be related in a different way with respect to negative emotions to personality functioning. The specific difficulty to accept positive emotional states in a non-judgmental way should determine specific responses not only on social and emotional functioning but also on the ability to adopt coping strategies and alternative adjustment responses, thus impacting academic and extra-academic lives (Menozzi et al., 2016; Dobos et al., 2021).

Despite literature emphasized the COVID-19 crisis in increasing emotion dysregulation and pathological personality domains (Zemestani et al., 2021; Waterschoot et al., 2022), no differences in pre- and post-pandemic groups were revealed, except for Detachment which surprisingly obtained lower scores in the post-pandemic condition compared to pre-pandemic one. A speculative explanation may be that restrictions requiring students to spend most of their time indoors with family members could strengthen family relationships by reducing their introversion and social isolation. However, this finding should be explored in further studies.

The results concerning students from LA are in line with the literature describing the psychological correlates of university students’ academic performance (Richardson et al., 2012; Honicke and Broadbent, 2016). Students, that are more likely to attribute the causes of successes or failures to their efforts or ability, are less prone to attribute those to task characteristics, luck, and help from other people. Moreover, choices between the two internal or among the external causes are more independent. Considering the beliefs, higher the level of effort attributions and higher the level of confidence in their own intelligence and mastery learning goals (Richardson et al., 2012); higher the level of luck attributions and lower the level of confidence in their own intelligence (Rotter, 1966). Results also showed that anxiety and resilience are associated with students’ beliefs more than with their causal attributions. It should be noted that the motivational components significantly contribute to academic achievement (e.g., Mega et al., 2014) and that anxiety and resilience were associated with the ineffective and the effective use of learning strategies (De Beni et al., 2014). These results corroborate the pattern of relationships among motivational aspects found in the aforementioned studies. Moreover, in line with the literature, they confirm the negative impact of the COVID-19 crisis on the well-being of students who more frequently reported higher levels of anxiety (e.g., Busetta et al., 2021). Together these findings suggest that an intervention aimed to promote internal causal attributions and reduce dysfunctional beliefs by modulating anxiety and resilience may effectively support students.

Overall, our findings show the importance of paying attention to characteristics such as emotion regulation, self-confidence, and learning motivation in promoting students’ wellbeing and academic careers and preventing the potential risks of severe psychological distress. Indeed, empowerment programs have been shown to reduce both depressive and anxiety symptoms in college students (Hart Abney et al., 2019). Lastly, although the expected negative effect of the pandemic period on the observed variables was only partially found, noteworthy is the increase in the number of students who have turned to the service.

### Limitations of the Study

Despite some promising results, several limitations of this study are worth mentioning.

First, the assessment tools (e.g., DERS, DERS-P, PID-5, AMOS, etc.) were mainly used to collect individual data as a support in the clinical counseling process, taking into account the specific problems underlying the search for help addressed to the two different intervention areas, ERA and LA. Therefore, the tools investigate different aspects and it was not possible to compare or aggregate the results of the ERA and LA groups. For the same reason, we did not carry out a post-test evaluation at the end of the counseling process. In the future, it will be important to ensure that all clients, both those accessing the ERA and those in the LA, will be assessed through the same tools in the initial assessment, as well as repeat the same assessment at the end of the consulting process in order to verify the effectiveness of the intervention.

In addition, the results about the impact of the COVID-19 crisis are difficult to interpret: on the one hand the pandemic situation *per se* probably caused the observed increase in demands for psychological help from university students, especially when reporting high levels of anxiety; on the other hand, the transition from the mode of delivery from presence to distance could have contributed to generate some counterintuitive effects or make less evident potential changes in some domains of personality. Further research is needed to distinguish the direct effects of the pandemic from changes related to the different ways of delivering services.

## Data Availability Statement

The original contributions presented in the study are included in the article/supplementary material, further inquiries can be directed to the corresponding author.

## Ethics Statement

Ethical approval was not provided as the data from this pilot study (which are presented in this brief research report) was collected primarily to verify the functioning of the counseling service and to be used clinically in counseling practice. It should be noted that all students attending the counseling service are required to read and sign an information sheet (see additional file) containing the following sentence “All the information provided will be used for research purposes only, the identity will not be disclosed and no data will be provided to third parties.” The patients/participants provided their written informed consent to participate in this study.

## Author Contributions

CSP and MZ contributed to conception and design of the study and wrote the sections Introduction and Discussion. CP wrote the first draft of the manuscript. PM and AA collected the data and organized the dataset. MCU and FB performed the statistical analysis and wrote the sections Methods and Results. All authors contributed to manuscript revision, read, and approved the submitted version.

## Conflict of Interest

The authors declare that the research was conducted in the absence of any commercial or financial relationships that could be construed as a potential conflict of interest.

## Publisher’s Note

All claims expressed in this article are solely those of the authors and do not necessarily represent those of their affiliated organizations, or those of the publisher, the editors and the reviewers. Any product that may be evaluated in this article, or claim that may be made by its manufacturer, is not guaranteed or endorsed by the publisher.

## References

[B1] Abdi R. Pak R. (2019). The mediating role of emotion dysregulation as a transdiagnostic factor in the relationship between pathological personality dimensions and emotional disorders symptoms severity. *Pers. Individ. Differ.* 142 282–287. 10.1016/j.paid.2018.09.026

[B2] Adamo S. M. G. Sarno I. Preti E. Fontana M. R. Prunas A. (2010). Brief psychodynamic counseling in a university setting. *Proc. Soc. Behav. Sci.* 5 1151–1159. 10.1016/j.sbspro.2010.07.252

[B3] Aldao A. Nolen-Hoeksema S. Schweizer S. (2010). Emotion-regulation strategies across psychopathology: A meta-analytic review. *Clin. Psychol. Rev.* 30 217–237. 10.1016/j.cpr.2009.11.004 20015584

[B4] Bandura A. (1997). *Self-Efficacy: The Exercise of Control.* New York: Freeman.

[B5] Busetta G. Campolo M. G. Fiorillo F. Pagani L. Panarello D. Augello V. (2021). Effects of COVID-19 lockdown on university students’ anxiety disorder in Italy. *Genus* 77:25. 10.1186/s41118-021-00135-5 34658399 PMC8502092

[B6] Cornoldi C. De Beni R. Fioritto C. (2003). “The assessment of self-regulation in college students with and without academic difficulties,” in *Advances in Learning and Behavioral Disabilities: Identification and Assessment*, eds Scruggs T. E. Mastropieri M. A. (Oxford: Elsevier/JAI), 231–242. 10.1037/cou0000102

[B7] De Beni R. Moé A. (1995). *Questionario di Attribuzione, Attribuzione Delle Cause di Successo/Fallimento in Compiti Cognitivi* (*Attribution Questionnaire, Attribution of Successes/Failures in Cognitive Tasks*). Firenze: Giunti OS Psychometrics.

[B8] De Beni R. Zamperlin C. Meneghetti C. Cornoldi C. Fabris M. Tona G. D. M. (2014). *Test AMOS - Abilità e Motivazione Allo Studio: Prove Di Valutazione e Orientamento per La Scuola Secondaria Di Secondo Grado e L’università* (*Ability and Motivation to Study: Evaluation and Vocational Tests for the Secondary School and University).* Trento: Nuova Edizione; Edizioni Centro Studi Erickson.

[B9] Denzine G. Brown R. (2015). “Motivation to Learn and Achievement,” in *Media Rich Instruction*, ed. Papa R. (Cham: Springer), 10.1007/978-3-319-00152-4_2

[B10] Dobos B. Piko B. F. Mellor D. (2021). What makes university students perfectionists? The role of childhood trauma, emotional dysregulation, academic anxiety, and social support. *Scand. J. Psychol.* 62 443–447. 10.1111/sjop.12718 33742444

[B11] Dupeyrat C. Mariné C. (2005). Implicit theories of intelligence, goal orientation, cognitive engagement, and achievement: A test of Dweck’s model with returning to school adults. *Contemp. Educ. Psychol.* 30 43–59. 10.1016/j.cedpsych.2004.01.007

[B12] Fossati A. Krueger R. F. Markon K. E. Borroni S. Maffei C. (2013). Reliability and validity of the Personality Inventory for DSM-5 (PID-5) predicting DSM-IV personality disorders and psychopathy in community-dwelling Italian adults. *Assessment* 20 689–708. 10.1177/1073191113504984 24065702

[B13] Giromini L. Velotti P. De Campora G. Bonalume L. Zavattini G. C. (2012). Cultural adaptation of the difficulties in emotion regulation scale: reliability and validity of an Italian version. *J. Clin. Psychol.* 68 989–1007. 10.1002/jclp.21876 22653763

[B14] Gratz K. L. Roemer L. (2004). Multidimensional assessment of emotion regulation and dysregulation: development, factor structure, and initial validation of the difficulties in emotion regulation scale. *J. Psychopathol. Behav. Assess.* 26 41–54. 10.1080/02699931.2021.2000370 34775912 PMC8860885

[B15] Hart Abney B. G. Lusk P. Hovermale R. Melnyk B. M. (2019). Decreasing depression and anxiety in college youth using the Creating Opportunities for Personal Empowerment Program (COPE). *J. Am. Psychiatric Nurses Assoc.* 25 89–98. 10.1177/1078390318779205 29865903

[B16] Honicke T. Broadbent J. (2016). The influence of academic self-efficacy on academic performance: a systematic review. *Educ. Res. Rev.* 17 63–84. 10.1016/j.edurev.2015.11.002

[B17] Karaman M. A. Eşici H. Tomar I. H. Aliyev R. (2021). COVID-19: are School Counseling Services Ready? Students’ Psychological Symptoms, School Counselors’ Views, and Solutions. *Front. Psychol.* 12:647740. 10.3389/fpsyg.2021.647740 33868121 PMC8044295

[B18] Mega C. Ronconi L. De Beni R. (2014). What makes a good student? How emotions, self-regulated learning, and motivation contribute to academic achievement. *J. Educ. Psychol.* 106 121–131. 10.1037/a0033546

[B19] Menozzi F. Gizzi N. Tucci M. T. Patrizi N. Mosca M. (2016). Emotional dysregulation: the clinical intervention of Psychodynamic University Counselling. *J. Educ. Cult. Psychol. Stud*. 1 169–182. 10.7358/ccps-2016-014-meno

[B20] Muzi S. Sansò A. Pace C. S. (2021). What’s Happened to Italian Adolescents During the COVID-19 Pandemic? A Preliminary Study on Symptoms, Problematic Social Media Usage, and Attachment: relationships and Differences with Pre-pandemic Peers. *Front. Psychiatry* 12:590543. 10.3389/fpsyt.2021.590543 33986698 PMC8110826

[B21] Pollock N. C. McCabe G. A. Southard A. C. Zeigler-Hill V. (2016). Pathological personality traits and emotion regulation difficulties. *Pers. Individ. Differ.* 95 168–177. 10.1016/j.paid.2016.02.049

[B22] Ponzoni S. Beomonte Zobel S. Rogier G. Velotti P. (2021). Emotion dysregulation acts in the relationship between vulnerable narcissism and suicidal ideation. *Scand. J. Psychol.* 62 468–475. 10.1111/sjop.12730 33956346 PMC8360132

[B23] Richardson M. Abraham C. Bond R. (2012). Psychological correlates of university students’ academic performance: A systematic review and meta-analysis. *Psychol. Bull.* 138 353–387. 10.1037/a0026838 22352812

[B24] Rotter J. B. (1966). Generalized expectancies for internal versus external control of reinforcement. *Psychol. Monogr. Gen. Appl.* 80 1–28. 10.1037/h0092976 5340840

[B25] Schunk D. H. Pintrich P. R. Meece J. L. (2008). *Motivation in Education: Theory, Research, and Applications*, 3rd Edn. Upper Saddle River, NJ: Pearson/Merrill Prentice Hall.

[B26] Sheldon E. Simmonds-Buckley M. Bone C. Mascarenhas T. Chan N. Wincott M. (2021). Prevalence and risk factors for mental health problems in university undergraduate students: A systematic review with meta-analysis. *J. Affect. Disord.* 287 282–292. 10.1016/j.jad.2021.03.054 33812241

[B27] Supriyanto A. Hartini S. Irdasari W. N. Miftahul A. (2020). Teacher professional quality: counseling services with technology in Pandemic Covid-19. *Counsellia Jurnal Bimbingan dan Konseling* 10 176–189. 10.25273/counsellia.v10i2.7768

[B28] Thomas K. M. Yalch M. M. Krueger R. F. Wright A. G. C. Markon K. E. Hopwood C. J. (2013). The convergent structure of DSM-5 personality trait facets and five-factor model trait domains. *Assessment* 20 308–311. 10.1177/1073191112457589 22946103

[B29] Velotti P. Rogier G. Civilla C. Garofalo C. Serafini G. Amore M. (2020). Dysregulation of positive emotions across community, clinical and forensic samples using the Italian version of the Difficulties in Emotion Regulation Scale-Positive (DERS-Positive). *J. Forens. Psychiatry Psychol.* 31 555–570. 10.1080/14789949.2020.1776374

[B30] Waterschoot J. Morbée S. Vermote B. Brenning K. Flamant N. Vansteenkiste M. (2022). Emotion regulation in times of COVID-19: A person-centered approach based on self-determination theory. *Curr. Psychol.* 1–15. [Epub ahead of print]. 10.1007/s12144-021-02623-5 35039734 PMC8754525

[B31] Weiner B. (1985). An attributional theory of achievement motivation and emotion. *Psychological Review* 92 548–573. 10.1037/0033-295X.92.4.548 3903815

[B32] Weiss N. H. Forkus S. R. Contractor A. A. Schick M. R. (2018). Difficulties regulating positive emotions and alcohol and drug misuse: A path analysis. *Addict. Behav.* 84 45–52. 10.1016/j.addbeh.2018.03.027 29625262 PMC5975124

[B33] Zemestani M. Babamiri M. Griffiths M. D. Didehban R. (2021). DSM-5 pathological personality domains as vulnerability factors in predicting COVID-19-related anxiety symptoms. *J. Addict. Dis.* 39 450–458. 10.1080/10550887.2021.1889752 33691610

